# Dietary Plant Polyphenols: Effects of Food Processing on Their Content and Bioavailability

**DOI:** 10.3390/molecules26102959

**Published:** 2021-05-16

**Authors:** Leila Arfaoui

**Affiliations:** Department of Clinical Nutrition, Faculty of Applied Medical Sciences, King Abdulaziz University, P.O. Box 80324, Jeddah 21589, Saudi Arabia; larfaoui@kau.edu.sa; Tel.: +966-0126401000 (ext. 41612)

**Keywords:** plant polyphenols, food processing, phenolic content, bioavailability, bioaccessibility

## Abstract

Dietary plant polyphenols are natural bioactive compounds that are increasingly attracting the attention of food scientists and nutritionists because of their nutraceutical properties. In fact, many studies have shown that polyphenol-rich diets have protective effects against most chronic diseases. However, these health benefits are strongly related to both polyphenol content and bioavailability, which in turn depend on their origin, food matrix, processing, digestion, and cellular metabolism. Although most fruits and vegetables are valuable sources of polyphenols, they are not usually consumed raw. Instead, they go through some processing steps, either industrially or domestically (e.g., cooling, heating, drying, fermentation, etc.), that affect their content, bioaccessibility, and bioavailability. This review summarizes the status of knowledge on the possible (positive or negative) effects of commonly used food-processing techniques on phenolic compound content and bioavailability in fruits and vegetables. These effects depend on the plant type and applied processing parameters (type, duration, media, and intensity). This review attempts to shed light on the importance of more comprehensive dietary guidelines that consider the recommendations of processing parameters to take full advantage of phenolic compounds toward healthier foods.

## 1. Introduction

Recent progress in nutrition and medicine has metamorphosed the way healthcare is conceived and delivered. An international technology-driven revolution is driving this rapid change from traditional healthcare to precision medicine by establishing unprecedented research programs and networks that prioritize diseases’ prevention and health promotion mainly through lifestyle and diet-based approaches [1]. A recent emerging area of precision nutrition focusing on bioavailable and metabolizable proportions of ingested foods with claimed health benefits has been developed. In this context, plant-derived polyphenols have been associated with several health benefits and are considered bioactive dietary compounds [2]. Polyphenols are the largest group of dietary antioxidants known for their ability to scavenge free radicals, donate hydrogen atoms, electrons, or chelate metal cations [3]. Because of these mechanisms, they have protective and preventive effects against several non-communicable diseases (NCDs), including cardiovascular diseases (CVDs), cancer, and diabetes [4].

However, the health implications of dietary polyphenols are determined by their bioavailability to a great extent, which is defined as the fraction of polyphenols released from the food matrix, metabolized, absorbed, and able to impose its bioactivity on the target cells or tissues [5]. Several factors influence polyphenol bioavailability, including the initial content in foods, food matrix, gut microbiota, and food processing [6]. In fact, most fruits and vegetables are not usually consumed raw. Instead, they go through industrial or domestic processing steps (e.g., cooling, heating, drying, fermentation, etc.) that affect their content, bioaccessibility, and bioavailability.

The main purpose of food-processing techniques is to transform raw ingredients into food, or to transform food into other end-products suitable for human or animal consumption. Some specific objectives include extending the shelf life of ingredients and products by inactivating pathogens or contaminating microorganisms, enhancing the bioavailability of otherwise inaccessible nutrients, enabling variety in flavor, texture, or aroma of certain foods, and improving the nutrient profile [7]. Domestic and industrial processing affect phenolic compounds’ content, antioxidant capacity, bioaccessibility, and bioavailability in different ways. While many food-processing techniques may lead to phenolic compounds’ degradation, some others enhance their absorption and bioavailability [8,9]. The ⁠final polyphenol content and bioavailability in processed food depend, therefore, on factors such as the nature of the process, duration of treatment, and food matrix subjected to the processing technique [10].

Since dietary polyphenol consumption has been increasingly proposed as an effective measure in the primary prevention and management of NCDs, it is imperative to consider the effect of food processing on their content and bioavailability. This review aims to summarize the effects of the most common food-processing techniques, either domestic or industrial, on dietary polyphenol content, bioaccessibility, and bioavailability. To introduce the topic, a glance at polyphenol types, sources, health implications, and the concepts of bioavailability and bioaccessibility is provided.

## 2. Polyphenol Types and Sources

The largest antioxidant group present in the human diet is that of phenolic compounds, with more than 8000 different structures identified to date [11]. Plants produce these secondary metabolites in response to ultraviolet light or pathogen attacks [12]. The molecular structure is based on one or several aromatic rings and at least one hydroxyl (phenol) group. They can either have a simple structure (such as in the case of phenolic acids) or a complex structure (such as in the case of flavonoids).

Based on their molecular structure, phenolics are divided into five main groups: phenolic acids, flavonoids, stilbenes, coumarins, and tannins [9]. As phenolic acids and flavonoids were mostly investigated in studies reporting the association between food processing and polyphenol content and bioavailability, these two classes are discussed in greater detail.

### 2.1. Phenolic Acids

Phenolic acids are non-flavonoid polyphenols, and their typical representatives are hydroxybenzoic acids (e.g., gallic, *p*-hydroxybenzoic, vanillic, and syringic acids) and hydroxycinnamic acids (e.g., ferulic, caffeic, *p*-coumaric, chlorogenic, and sinapic acids). They exist in bound or free form in fruits and vegetables. Grains and seeds are particularly rich in bound phenolic acids, which are released after acid or alkaline hydrolysis or enzymatic reactions [11]. Good sources of phenolic acids are fruits (apples, cherries, berries, and their products, such as wine), vegetables (broccoli, lettuce, and tomatoes), legumes, cereals, and coffee beans [13].

### 2.2. Flavonoids

Flavonoids are a large group of polyphenols that typically contain two aromatic rings linked by a heterocycle. Their subclasses are distinguished by structural differences based on this heterocycle [14]. These subgroups include anthocyanins, flavan-3-ols, flavones, flavanones, and flavonols [11].

Flavones, flavonols, and flavanones are widely distributed in plants. Flavones and their derivatives flavonols, as well as their acylated products, represent the largest polyphenol subgroup [15]. Quercetin and kaempferol are, for example, the most commonly found flavonol aglycones, and they alone have approximately 300 different glycosidic combinations [15]. The most relevant flavone sources are citrus fruits, parsley, lettuce, and grapes, while flavanones are mostly present in citruses and products based on them [13]. Among all flavanones, hesperidin and naringenin are typical representatives. Good dietary sources of flavonols include plums, apples, onions, and blueberries, with kaempferol and quercetin being the main representatives [13].

Flavanols are a very complex group of polyphenols, which include compounds ranging from monomeric flavan-3-ols to polymeric proanthocyanidins. Proanthocyanidins are the precursors of anthocyanidins, which are produced under acidic conditions as a result of polymeric chain cleavage [11]. The most commonly identified monomeric flavanols in dietary sources are catechin, epi(gallo)catechin, and their gallates [13]. Catechins are mainly present in tea, grapes, red wine, cocoa, and chocolate [13].

Anthocyanidins are compounds responsible for the red, blue, and purple pigments in fruits and vegetables, flower petals, and some grain varieties, such as black rice. As they are mainly present as glycosides, they are commonly referred to as anthocyanins. Among the 31 currently known anthocyanidins, the most frequently identified in plants are cyanidin, delphinidin, and pelargonidin [16]. In fact, 90% of the structure of anthocyanins is based on these compounds and their derivatives [16]. Several factors influence the color of anthocyanins, such as pH and degree of hydroxylation. Depending on these factors, these phenolic compounds can either give blue, red, or purple colors to plants [11]. A wide range of plant-based foods are good sources of these phenolics, with grapes, red wine, berries, and some vegetables being typical representatives [13].

These diverse polyphenol sources and types discussed above are valuable, and therefore it is worth investigating their bioavailability and health benefits in light of the scientific literature available so far.

## 3. Polyphenols’ Health Benefits Related to Chronic Diseases

**Figure 1:**
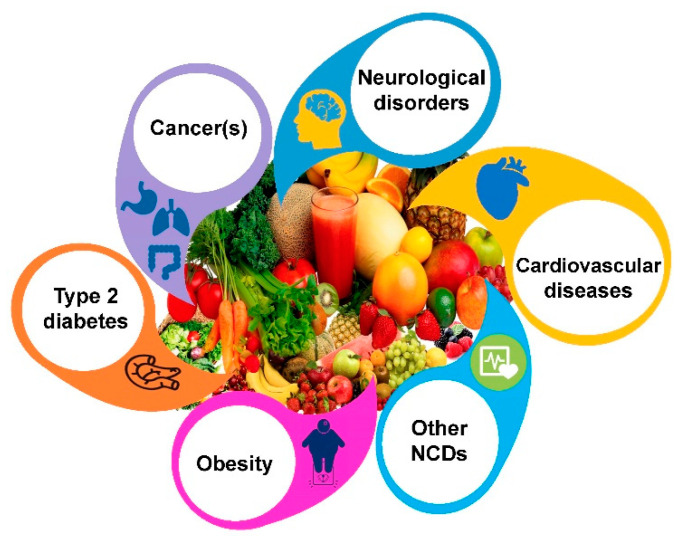
Main health benefits of polyphenols related to chronic diseases.

Oxidative stress and inflammation are common pathways that drive the progression of many NCDs. As polyphenols impair these processes [17], incorporating higher doses of these compounds into the human diet may be a suitable tool for primary prevention, lowering the incidence and delaying the onset of several chronic diseases, including cardiovascular diseases, cancer, obesity, and neurological disorders (Figure 1).

### 3.1. Polyphenols Consumption and Their Effects on Obesity

Animal, in vitro, and human studies have shown that dietary anti-inflammatory and antioxidative compounds may increase thermogenesis and energy expenditure, and decrease inflammation and oxidative stress, thereby facilitating weight loss and/or reducing the rate of metabolic conditions [18]. Some polyphenols with anti-obesity properties are tea catechins, specifically epigallocatechin-gallate (EGCG). In vitro studies on isolated EGCG or green tea extracts have reported their potential to inhibit preadipocyte differentiation, decrease adipocyte proliferation, and induce apoptosis. They also suppress lipogenesis and promote lipolysis and beta-oxidation processes [19]. A preclinical animal study has reported that EGCG and green tea extract facilitate weight loss by lowering adipose mass in mice fed a high-fat diet [20]. Other polyphenols with anti-obesity properties include anthocyanins, compounds with great antioxidant [21] and anti-inflammatory potential [22]. Few human clinical trials have assessed the obesity-related effects of anthocyanin-rich foods; however, the available data suggest that black soybean and red orange juice may effectively reduce markers of inflammation and metabolic disease in overweight people [23,24].

### 3.2. Polyphenols Consumption and Their Effects on Cardiovascular Diseases

Oxidative stress has been linked to endothelial dysfunction, which triggers the onset of early atherosclerosis and CVD. Thus, strategies that aim to reduce oxidative stress and inflammation may be promising to combat CVD-related disorders. In addition, polyphenols can increase NO synthase activity [25], which in turn positively affects flow-mediated dilatation (FMD). Human trial data suggest that oligomeric proanthocyanidins are an efficient dietary intervention for improving plasma lipid profiles and anti-atherogenic components of plasma, as observed in 70 hyperlipidemic subjects [26]. A 6-week supplementation with 162 mg of onion peel quercetin per day was reported to improve hypertensive patients’ systolic blood pressure [27]. However, a daily dose of 100 mg quercetin for 12 weeks did not affect blood pressure in 72 healthy overweight and obese subjects, whereas it positively affected endothelial function measured as FMD and circulating endothelial progenitor cell counts [28]. When the findings from randomized controlled trials (RCTs) of polyphenol supplementation were meta-analyzed, it was concluded that polyphenols could exert beneficial effects on LDL-c, HDL-c, FMD, and both systolic and diastolic pressure, thus supporting their implications in primary CVD prevention [29].

### 3.3. Polyphenols Consumption and Their Effects on Type 2 Diabetes (T2D)

Polyphenols and polyphenol-rich products can also ameliorate the risk of T2D. Polyphenols are involved in attenuating postprandial glycemic responses, fasting hyperglycemia, and improving insulin secretion and sensitivity. These implications might be explained by their ability to inhibit the digestion of carbohydrates and intestinal glucose absorption, stimulate insulin secretion, modulate the liver-related release of glucose, and trigger both gene expression and cell signaling pathways [30]. Many studies have investigated the efficacy of polyphenol supplementation at the onset of T2D. One meta-analysis of observational studies found that a diet rich in polyphenols (specifically flavonoids) may serve as a tool to prevent the onset of T2D [31]. Regarding specific food groups, tea consumption was reported to be inversely associated with the onset of T2D. A dose–response meta-analysis of cohort studies suggests that consuming 4–6 cups of tea per day may decrease T2D risk by up to 15% [32]. Other polyphenol-rich food groups linked with a reduced risk of T2D include fruits, specifically berries and yellow vegetables [33,34].

### 3.4. Polyphenols’ Consumption and Their Effects on Cancer

Polyphenol supplementation can hamper cancer progression, probably because of their involvement in cancer cell apoptosis. They were also shown to modulate cell cycle signaling and promote cell defense systems [35]. Epidemiological studies have shown that dietary polyphenol intake is linked with lower cancer incidence. For example, one Canadian case-control study observed that high dietary flavonoid consumption could reduce lung cancer occurrence [36], while a Korean study confirmed a similar relationship with gastric cancer [37]. Clinical trials also support the notion that polyphenol compounds may exert beneficial effects on disease progression [38,39]. A meta-analysis of 165 prospective and case-control studies did not confirm the association between total flavonoid intake and cancer risk. However, the subgroup analysis confirmed that a higher intake of some flavonoid classes could lower the risk of some cancers, specifically colon, lung, and stomach ulcers [40].

### 3.5. Polyphenols’ Consumption and Their Effects on Neurological Disorders

Finally, the antioxidant properties of polyphenols are important for improving brain health and function. By scavenging free radicals, they facilitate the reduction of brain cell damage [41]. Using animal models with neurocognitive dysfunction, it has been observed that physiological doses of flavonoids may reduce the accumulation of neuropathological proteins and improve synaptic plasticity [41]. Observational studies and RCTs suggest that dietary polyphenols, such as those present in berries, grapes, cocoa, and green tea, may modulate processes related to cognitive health [42]. It was also reported that resveratrol supplementation could enhance both cerebrovascular function and cognition in postmenopausal women, implying that this polyphenol’s presence in the diet may improve cognitive function in this specific population [43]. A meta-analysis study found that polyphenol consumption (that is, those present in Ginkgo biloba, resveratrol, and soy isoflavones) did not significantly affect the scores reflecting global cognitive functions; however, they improved those scores related to verbal learning, memory, and executive functions, while not affecting attention-related scores [29].

## 4. Bioavailability of Polyphenols

Along with the polyphenol content in foods and their distribution in plants, bioavailability is an essential factor that directly influences and determines the biological function of polyphenol-rich-food consumption. A distinction between bioaccessibility and bioavailability should be made. Bioaccessibility refers to the fraction that mobilizes from the food matrix and reaches the gastrointestinal system, undergoing digestion, absorption into intestinal epithelial cells, and metabolic changes in the intestine and liver [44]. This definition does not include their utilization in target cells or tissues, such as in the case of bioavailability [45]. Bioavailability refers to a fraction released from the food matrix, subjected to digestion, absorption, metabolism in the liver and intestine, and its further distribution to target tissues and cells where it imposes its bioactivity and exerts positive health-related effects. It is complicated to measure bioactivity owing to practical (and ethical) issues; therefore, bioavailability definitions today do not include bioactivity [46].

### 4.1. Bioavailability and Bioaccessibility Assessment

As seen in the previous section, evaluating bioaccessibility is usually carried out by using in vitro methods that simulate digestion, and in some cases, the simulation of uptake by Caco-2 cells is also included [47]. By contrast, bioavailability is measured in vivo as the change in plasma levels (in humans or animals) of that specific compound after an acute or chronic administration of the food matrix containing the investigated compound [48].

According to published literature, analyses of bioavailability and bioaccessibility of polyphenols have been carried out by using both in vitro and in vivo models. The most utilized in vitro method is the static gastrointestinal model, and several modifications have been applied to it, such as the addition of experiments that simulate the oral phase during digestion or colonic fermentation. One of the models that allow the implementation of colon fermentation experiments is the dynamic gastric model, which has been frequently applied to study polyphenol bioavailability. In addition to in vitro models, in vivo assessments have been performed in rats, pigs, and dogs, and the number of clinical trials in humans has been rapidly increasing during the last decade [49].

### 4.2. Factors Influencing Polyphenols’ Bioavailability

**Figure 2:**
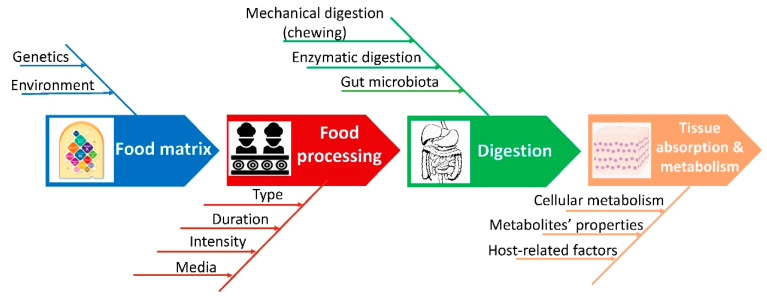
Main factors affecting dietary plant polyphenols content and bioavailability.

It is challenging to conduct bioavailability studies because several different factors influence it (Figure 2). Some of them are external, such as environmental factors (rainfall, sun exposure, soil type, etc.) or the degree of ripeness. The composition of the food matrix and the interaction between polyphenols and other dietary components (proteins, fat, carbohydrate, and fiber), as well as the tendency of polyphenols to build complexes with proteins, should be considered when assessing their bioavailability [10].

The chemical structure of a specific polyphenol determines its fate during digestion in a significant manner. Dietary polyphenols mostly exist as polymers or glycosides, containing a glycone part (the sugar group) and an aglycone part (the polyphenol). Most phenolic compounds resist acidic conditions during gastric digestion [50], and as they cannot be absorbed in their native form (except for anthocyanins), the intestinal enzymes or the colonic microbiota hydrolyze them prior to absorption. Thus, the degree of absorption of polyphenols in the intestine greatly depends on the chemical structure and the type of sugar present in the glycosylated form [10].

The host that ingests food containing polyphenols also influences their metabolism. Specifically, there are two important factors: the intestinal factors and the systemic factors. In fact, the ingested polyphenols undergo several biochemical changes in the small intestine mainly through glycoside hydrolyzation to allow their absorption [10]. The non-absorbed fraction of polyphenols will transit to the colon, where they are transformed by the colonic microflora into other bioactive phenolic metabolites, followed by structural modifications mainly in the liver prior entering the blood stream [34,51,52,53]. Besides the intestinal factors, these biochemical transformations of polyphenols in the digestive tract and the type and proportions of their derivative metabolites depend also on the host systemic factors, such as gender, age, presence of pathologies, and genetics [10].

Finally, the processing techniques applied in domestic or industrial settings influence the final polyphenol content in the food and thus its bioavailability (Figure 2). The studies investigating these processing-related effects are discussed in the fifth section of this review.

### 4.3. Polyphenols’ Metabolism Pathway during Digestion

Some phenolic compounds are absorbed in the small intestine and those that are not are metabolized in the colon, where glycosides are hydrolyzed into aglycones and further degraded into simple phenolic acids [51,54]. These changes depend on their chemical structure. For example, anthocyanidins are highly unstable and are absorbed as glycoside-anthocyanins [55]. As they are highly susceptible to gastric conditions, a very low amount of anthocyanins is bioavailable (approximately 0.1% of the intake) [56]. It is generally observed that those phenolics that are absorbed in the colon have lower bioavailability rates than those that undergo these processes in the intestine [50]. The bioavailability of proanthocyanidins is determined by the degree of polymerization. Most of these phenolics pass through the small intestine to the colon, where the colon microbiota modifies them [57]. Those with a lower degree of polymerization undergo more intense modifications, while those with a higher degree form complexes with macromolecules that disables their further modifications and absorption [58]. Diverse phenolic acids and flavan-3-ols are produced by the reactions between proanthocyanidins and the colonic microbiota, and these products undergo further absorption in the colon [58]. In the case of phenolic acids, the small intestine absorbs one part, and the colon absorbs the other [14].

After absorption by epithelial cells, phenolic acids or colonic metabolites reach the liver through the portal vein, and they undergo conjugation processes described as phase II metabolism (methylation, sulfation, and glucuronidation). Some conjugation processes occur in the small intestine; however, the majority occurs in the liver [10]. Almost all plasma phenolics are products of phase II metabolism, and these metabolites determine the biological activity of ingested dietary polyphenols [45]. These extensive metabolic changes produce several metabolites from a single polyphenol. Therefore, the compounds that reach the target cells and tissues where they impose their bioactivity are very different from the original form present in food, chemically, biologically, and in many other ways.

## 5. Food-Processing Techniques and Their Effects on Polyphenols Content and Bioavailability

Vegetables and fruits are a main source of phenolic compounds. For example, several fruits as apple, berries are very rich in polyphenols with more than 200 mg per 100 g of fresh fruits [59]. However, the content and the bioavailability of these phenolic compounds are influenced by the food-processing technique(s) applied. For instance, and since many food-processing methods involve heat treatment, it is believed that higher temperatures may lead to detrimental changes in fruits and vegetables in terms of their nutritional profile; however, some studies observed quite the opposite [8]. A detailed analysis of 161 polyphenols and their food-processing changes reported that domestic cooking induced notable losses in polyphenols, with great variability between the foods. It was also observed that the food that was investigated was often a more important factor than the process employed, thus highlighting the importance of the food matrix [60]. Many other factors influence the fate of polyphenolic compounds during food processing, and these relationships are briefly presented in the following subsections, depending on the type of food-processing technique used at both industrial and domestic sittings (Appendix A Table A1 and Table A2).

### 5.1. Thermal Processing

#### 5.1.1. Heat Treatment

Heat treatments are commonly applied in food processing in both domestic and industrial settings. These treatments include boiling, frying, steaming, baking, stewing, and roasting in traditional, microwave, and steam ovens. Heat is also utilized in other traditional transformation processes such as toasting, coffee roasting, drying, canning, pasteurization, and sterilization.

The fate of polyphenols during thermal processing largely depends on the method applied. The heat ruptures cell walls, enabling the bound phenolics to mobilize to other parts of the plant, enhancing their availability [61]. However, at the same time, they are more prone to oxidation, and some are more or less thermostable.

Studies applying thermal treatments have shown that boiling causes the most detrimental polyphenol composition changes in treated samples. Steaming and frying can preserve higher amounts of these compounds. The underlying reason might be that phenolics are water-soluble, and during the boiling process, they leak into the surrounding medium (water). As heat treatment decomposes the tissue, it enables the migration of cellular components and nutrients into the boiling water [62]. It has been proposed that polar media (water) might be responsible for higher losses due to polyphenol solubility in water, while nonpolar media (oil) would extract lower amounts of lipid-insoluble polyphenols [63].

The detrimental effects of boiling were observed in the case of onions and asparagus. A 60 min boiling process led to a 20.6% and 43.9% loss in overall flavonol content in onions and asparagus, respectively. Notable changes were also observed in the antioxidant capacities [64]. This was confirmed in another study, in which boiling for 1 h induced 53% and 44% degradation of quercetin diglucoside and monoglucoside in onion bulbs, respectively, while boiling for 30 min, frying, and roasting (in microwave and oven) caused less severe changes [65]. The higher degradation of quercetin derivatives might have occurred because of the difference in the surrounding medium, where water as a polar medium led to a higher extraction of these compounds than oil and air. However, this study also points to the polyphenol class being an important determinant—in the case of anthocyanins (cyanidin 3-glucoside, cyanidin 3-laminaribioside, and cyanidin 3-(6″-malonylglucoside) and cyanidin 3-(6″-malonyl-laminaribioside), frying caused the most severe effects, followed by boiling and roasting [65].

Water volume can also influence the degree of polyphenol alteration during heat treatment. Cooking in smaller volumes resulted in higher phenolic content in zucchini (specifically rutin), beans (rutin and quercitrin), and carrots (chlorogenic acid). Accordingly, cooking in smaller water volumes yielded lower phenolic concentrations in the surrounding water than cooking in larger volumes [66].

Musilova et al. explored variety as an influencing factor that modulates the relationship between food processing and polyphenol alterations. Four sweet potato varieties were subjected to four different heat treatments (boiling, steaming, microwaving, and baking). In general, heating had adverse effects on phenolic acid levels. Boiling was shown to have the most detrimental effect: it lowered the levels of chlorogenic acid by 29% (in the 414-purple variety from Slovakia), neochlorogenic acid by 69% (in the 414-purple variety from Croatia), and trans-ferulic acid by 29% (in the Beauregard variety from Croatia). In contrast, all these treatments increased the levels of total polyphenols, total anthocyanins, and total antioxidant activity, mostly in all analyzed samples [67].

Different boiling periods yielded different polyphenol profiles in foods. For example, blanching (short-interval boiling) of kale leaves led to a 51% decrease in polyphenol content (phenolic acids, flavones, flavonols, and anthocyanins), the lowest of which was observed in the case of caffeic acid (28%) and the highest in ferulic acid (55%). However, cooking for a longer time caused more severe damage, resulting in a 73% decrease in the total polyphenol content [68].

Chumyam et al. highlighted the role of the medium in their study by comparing the utilization of water as a medium (in the case of boiling and steaming) and no medium (in the case of microwaving). All procedures were conducted in 5, 10, and 15 min. All treatments significantly increased antioxidant capacity and total phenolic content in purple skin eggplant compared with raw samples. Boiling yielded samples with the lowest levels of total polyphenols [69]. Of all the methods investigated, 10-min microwaving resulted in the highest total phenolic level and highest antioxidant capacity. In addition, 10-min microwaving proved to be the best method for enhancing the antioxidant capacity of eggplant fruits [69]. The authors proposed that phenolic and antioxidant compounds activated by microwaving remained in eggplant fruits, while cooking by boiling and steaming caused their leakage (either directly or indirectly) into water.

High-temperature regimes are also applied to preserve food by inactivating enzymes and pathogen microbes, with sterilization and pasteurization as two commonly applied treatments. In white beans, sterilization significantly decreased the total polyphenol content after 4 and 12 months of storage (by 30 and 46%, respectively). However, these findings may be biased by the effects of storage. This was also observed in condensed tannins; after 12 months of storage, the concentration of these compounds was lower by 30% [70]. Pasteurization of apple juice by conventional high-temperature/short-time treatment also led to notable losses in total polyphenols (by 32.3%). However, pasteurization with pulsed electric field treatment reduced their levels by only 14.9% [71].

Studies have also assessed the effects of thermal processing treatments on the bioavailability of dietary polyphenols. Domestic cooking of cherry tomatoes increased the bioavailability of naringenin and chlorogenic acid in five test subjects [72]. Mechanical and heat treatments applied during tomato sauce production also enhanced the plasma concentration and urinary excretion of naringenin glucuronide. This was observed in a randomized controlled trial in which eight healthy volunteers ingested either tomato sauce or raw tomatoes [73]. These data confirm that heat treatment enhances the mobilization of polyphenol bioactive compounds from the food matrix, making them more bioavailable.

The type of heat treatment is an important determinant of polyphenol bioavailability. When cassava samples were subjected to different cooking methods, the highest total phenolic content was observed in steaming, followed by microwaving and boiling. These cooking methods similarly affected their availability in the subjects, although with only slight differences, steamed samples showed a 74.5% bioavailability, while boiled and microwaved samples obtained similar values (72.9% for boiled and 72.7% for microwaved samples) [74]. Streaming was the best heat method, probably due to the indirect exposure to the polar medium (water); thus, the polyphenols were retained to a higher degree in the plant.

The form in which phenolics are present is a well-recognized factor, as observed in carrots when exposed to heating in a microwave oven for 12 min. Although it did not affect anthocyanin bioavailability, it increased the relative urinary recovery of non-acylated anthocyanins without affecting the acylated anthocyanins. The authors proposed that the acylation of anthocyanin derivatives is an important factor when investigating their bioavailability [75].

Finally, Rodriguez-Mateos et al. [76] investigated how baking affected the content and bioavailability of blueberry polyphenols, namely anthocyanins, procyanidins, and phenolic acids. This RCT involving 10 healthy subjects used a baked product containing blueberries, an unprocessed blueberry drink, and a matching control baked product. Processing induced no significant changes in the total polyphenolic content; however, it significantly lowered the amounts of anthocyanins (−42%), increased the levels of chlorogenic acid (23%), and significantly enhanced the concentrations of flavanol dimers and trimers (36 and 28%, respectively). By determining the plasma levels of 22 metabolites assessed individually after ingestion of the test products, the authors observed that the blueberry baked product induced mainly an increase in four metabolites (m-hydroxyphenylacetic, ferulic, isoferulic, and hydroxyhippuric acids) and a decrease infour others (hippuric, benzoic, salicylic, and sinapic acids) compared with the blueberry drink, without affecting the bioavailability of the total phenolic levels [76].

#### 5.1.2. Canning

Canning is a process that aims to produce commercially sterilized and microbiologically safe products by applying heat treatment. Canned food products can be produced by using retorts, pasteurizers, or heat exchangers. The main aim of heat treatment is to eliminate pathogens or microorganisms that can cause contamination, while metal containers, glass jars, and retort pouches are used to prevent spoilage by new microorganisms. After heating, the canned product is cooled and stored at room temperature to maintain the stability and integrity of both the food and the container/jar used [77].

Canning is reported to reduce total phenolic and flavonoid contents, mostly because phenolic compounds leach into the surrounding medium (brine or syrup) [78]. This extensive leaching results from the applied heat treatment, which disintegrates the cells and tissues, enabling the migration of polyphenols into the medium.

There is also a difference in how the canning process is carried out and the corresponding conditions. One study examined the differences in the effects of domestic and industrial canning on apricots. Thermal processing imitating industrial conditions induced a higher loss of total phenolics (13–47%), while domestic processing led to lower losses (2–33%). This is proposed to be the consequence of the difference in temperature regimes, the more intensive one being employed in industrial processing. Procyanidins were similarly affected: the loss in domestic treatments was 2.4%, and that in industrial treatments was 44%. Phenolic acids (hydroxycinnamic acids, 3-*O*-caffeoylquinic acid, and 5-*O*-caffeoylquinic acid) exhibited a significant decline in both thermal processes; however, the greater loss was observed in industrial canning [79].

Interestingly, it has also been reported that canning may lead to the production of some desirable compounds that are not naturally present in raw foods. Investigation of canned peanuts showed that the retorting process damaged some phenolic compounds (resveratrol, caffeic acid, and catechin); however, genistein (a phytoestrogen belonging to the class of soy isoflavones), which was not measured in raw, was quantified in the canned peanut [80].

#### 5.1.3. Drying

Drying is a preservation process that aims to reduce the moisture content of food by using heat and mass transfer [81]. As fruits and vegetables are prone to microbial and biochemical spoilage due to their high water content (which can be more than 80%), drying reduces the water activity and makes a stable end-product with an extended shelf life. Some of these methods include vacuum-drying, solar-drying, air-drying, and freeze-drying.

Since drying involves heat treatment, the temperature regime affects the degree of degradation of dietary polyphenols, as observed in jujube fruits. Jujube fruits were subjected to different drying procedures: vacuum–microwave (480, 120 W), hot air (70, 60, and 50 °C), and combined methods such as pre-drying and finish-drying (60 °C + 480/120 W). Freeze-drying (the control treatment) was the most efficient method for preserving total phenolic content, while hot-air-drying caused the greatest loss in these compounds; the highest losses were observed after applying the highest temperature. The polyphenol content was less affected when the methods were combined. Similarly, antioxidant activity was the highest in freeze-dried samples; high air temperature (60 or 70 °C) caused detrimental changes to antioxidant compounds [82]. In cocoa beans, total polyphenol content decreased with an increase in temperature, with the highest decrease being observed at 60 °C, and the lowest at 40 °C. These detrimental processes may be the result of oxidation and cellular destruction. Moisture content in beans can also be another limiting factor, as it affects the volatility of these compounds and the degree of polyphenol solution [83].

However, the variability of the effects caused by drying is also influenced by the variety and polyphenol class. One study assessed how two drying treatments—one involving air-drying at 55 °C and the other at 75 °C—affected two apricot cultivars. Interestingly, chlorogenic and neochlorogenic acid contents were observed to increase in the higher temperature regime (in one cultivar), and catechin levels showed the same trend in both cultivars. The authors proposed that polyphenol oxidase remained active to a great extent and that its activity affected the higher production of these two phenolic acids. However, the flavonol content decreased proportionally with the increase in temperature, as well as the content of epicatechin [84].

In some cases, the lowest temperature was not the best solution for retaining high polyphenol levels in the samples but optimizing the temperature and drying period. In a study on cocoa beans at three temperature regimes (drying at 60, 70, and 80 °C), the maximum concentration of total phenolics was reported to be in samples that were dried at 70 °C, and their levels decreased as the heating procedure was prolonged [85]. In another study, the researchers subjected several berry fruits (raspberry, boysenberry, redcurrants, and blackcurrant fruit) to drying at 50 °C for 48 h, 65 °C for 20 h, or 130 °C for 2 h until a moisture content below 15% was reached. Drying at 65 °C was the best method in terms of the total polyphenol content and radical scavenging activity. Higher temperatures and longer regimes proved to be detrimental to the polyphenols in berries. Specifically, anthocyanin levels were similar in berries dried at 50 and 65 °C. However, temperatures above 100 °C caused the most detrimental effects on all the analyzed parameters [86].

According to the available literature, drying procedures may be used to obtain products with more bioavailable polyphenols. However, the final effect depends on the specific polyphenol class or the individual compounds investigated and the temperature regime. Kamiloglu et al. investigated the effect of home processing on the availability of total phenolics and flavonoids in tomatoes by employing an in vitro gastrointestinal digestion model. They observed that oven drying (70 °C for 36 h) yielded a two-fold higher bioavailability of total polyphenols; however, this was not the case with total flavonoids [87]. In another study, Kamiloğlu and Capanoglu (2013) observed that an 8 d sun-drying procedure at 30 °C positively affected the bioaccessibility of chlorogenic acid, while adversely affecting rutin and anthocyanins (cyanidin-3-glucoside (C3G) and cyanidin-3-rutinoside) in yellow and purple figs, which were undetectable after in vitro digestion [88]. Finally, when conventional hot-air-drying in an oven (60 °C for 24 h) was compared with freeze-drying, the authors observed that oven-drying induced more favorable changes in pumpkin flour. The oven-dried samples had higher phenolics, bioaccessible phenolics and phenolic acids, and antioxidant activities [89].

### 5.2. Cold Processing

Freezing is a preservation technique that leads to the formation of water crystals and ice below the freezing temperature, thus slowing down biochemical and physicochemical reactions. Consequently, it stops the activity of most pathogenic microorganisms and enables longer shelf life of foods [90].

Chilling or cooling also reduces microbial and biochemical alterations in foods to maintain stability but to a lesser degree (the temperature range is between −1 and 8 °C). Cooling decreases the initial temperature of the food product and maintains its quality for a longer period [90].

The literature mainly points to these low-temperature regimes as non-invasive methods for preserving polyphenols in foods, as observed by Korus and Lisiewska, who concluded that freezing of either blanched or cooked kale leaves did not induce notable changes while freezing decreased the polyphenol levels by 3% and their antioxidative activity by 7% [68]. In addition, freezing at −30 °C of fresh red raspberries did not alter their antioxidant capacity or phenolic levels, measured as total phenolics, anthocyanins, lambertianin C, sanguiin H-6, and ellagic acid [91]. However, in some cases, these techniques can even enhance the content of polyphenols, such as in maqui fruits in which cooling (5 °C) and freezing (−20 °C) increased the total polyphenol concentration. The frozen samples had higher levels of anthocyanins, and the changes were variable. In addition, the freezing technique was better in terms of preserving these antioxidants, as they were present in these samples even after six months of storage [92].

One of the most important factors that affect the degree of dietary polyphenol preservation during food freezing is the freezing rate, as observed in a study that reported that slow-frozen strawberries had lower levels of monomeric anthocyanins than quick-frozen ones [93]. Similarly, Poiana et al. observed that individual quick freezing had no significant effect on polyphenolic compounds (total phenolic content, total monomeric anthocyanins) in three berries: blueberry, red raspberry, and blackberry [94]. The proposed mechanism is based on the nature of crystal formation. Freezing is a process that leads to the formation of ice crystals, making solutes (such as anthocyanins) localized and relocating the water molecules in the cell structure. Quick freezing induces the formation of smaller crystals, and consequently, the cells undergo less damage during the freezing process. The thawing process will also lead to a lower phenolic loss in quick-frozen samples because less water migrates in the quickly frozen strawberries, and consequently, less polyphenols as well [93].

Literature on the effects of freezing/cooling on dietary polyphenol bioavailability is scarce. One study reported that freezing by immersion in liquid nitrogen and freeze-drying at −50 °C decreased the levels of total polyphenols and antioxidant activity in apples, both before and during in vitro gastric digestion. Raw apples, which served as control samples, retained the highest amount of polyphenols and exhibited small decreases in total polyphenol concentrations and antioxidant activity during in vitro gastric digestion [95]. As mentioned above, freezing can damage the food matrix due to ice crystal formation, especially in slow freezing. Consequently, polyphenols have been proposed to undergo higher extraction during digestion, which would enhance their bioavailability. At the same time, they are also more prone to oxidation and degradation, especially when food undergoes thermal treatment after freezing. Accordingly, it could be expected that freezing would have significant effects on polyphenol bioavailability; however, there are no data to address this question [45].

### 5.3. Biochemical Processing

#### Fermentation

Fermentation is a non-thermal process based on the activity of specific microorganisms and their enzymes that induce chemical alterations in food components, leading to a significantly transformed food product. Examples of such food products are fermented milk products (yogurt or cheese), bread, vegetal probiotic beverages, vinegar and alcoholic beverages. Today, these processes are well controlled and can yield products with desired characteristics by selecting an appropriate bacteria or yeast [96].

Fermentation is an affordable technique that transforms grains into edible foods by increasing their nutrient bioaccessibility and by positively affecting their antioxidant profile and activity in the end-products. Adebo et al. found higher levels of catechin, gallic acid, and quercetin in samples of fermented whole grain sorghum, while the total flavonoid content, total tannin content, and total phenolic content decreased [97]. The authors proposed that these decreases can be explained by the degradation and hydrolysis of the phenolic compounds, while the increase might be the consequence of fermentation by Lactobacillus strains. Another study reported an increase in total phenolic content in whole grain millet-koji, which might result from the mobilization of phenolic compounds from their bound form into a free form by the activity of the fermentation-produced enzymes [98].

Legumes have also been investigated as food classes in several studies. Eight commonly consumed legume varieties (black cow gram, mottled cowpea, speckled kidney beans, lentils, small rice beans, small runner beans, and two soya beans) were subjected to fermentation by naturally present bacteria and lactic acid bacteria. All fermented legumes had a high total phenolic content, probably owing to the biotransformation between bound and soluble phenolics. Thus, fermentation in legumes increases the bioavailability of polyphenols [99]. In four underutilized legumes (pigeon pea, Bambara groundnut, African yam bean, and kidney bean), a 4-day fermentation period increased the free soluble phenol content, whereas the bound phenolics were decreased. Free soluble phenolic compounds from fermented samples had a significantly higher reducing power, free radical scavenging ability, and inhibition of lipid peroxidation than the unfermented samples, confirming that fermentation led to enhanced antioxidant activity in the legumes [100]. Thus, in both cases, fermentation led to products with better nutrient profiles and antioxidant characteristics.

As observed in the case of heat treatment, the variety determines how fermentation affects polyphenol compounds as well. Fermentation of five different varieties of apple juice led to variable changes in the phenolic profile. In the three varieties, the levels of total phenolics remained the same after the process; however, in two of them, they decreased [101].

However, studies assessing the role of fermentation in improving red grape polyphenol bioavailability have indicated that fermentation may play a minor role. In a trial involving nine volunteers ingesting equal amounts of red wine and red grape juice (resulting in almost equal concentrations of anthocyanins), the authors observed higher urinary excretion of total anthocyanins in the case of juice (0.23%) than in wine (0.18%). They also reported that urinary recovery of five individual anthocyanins was different between the two grape products, without reaching significance in both. Thus, they suggested that these results might be because of the presence of ethanol in red wine [102]. This was not confirmed by Bub et al. [103], who compared the absorption of malvidin-3-glucoside between red grape juice, red wine, and dealcoholized red wine. The bioavailability of this compound was similar when volunteers consumed regular wine or those without alcohol; however, it was two-fold higher in the case of juice consumption. According to these results, it cannot be concluded that the ethanol produced by fermentation affects the accessibility of these red grape polyphenols.

Black tea is another fermented polyphenol-rich beverage widely consumed in the human diet. Both green and black teas are a great source of monomeric flavanols (catechins). The difference between these two types of tea is in the production process. Green tea is a product obtained by drying leaves of Camellia sinensis, while the production of black tea involves an additional fermentation step, resulting in the synthesis of oligomeric polyphenolic compounds from catechins. Thus, black tea contains lower levels of these flavanols than green tea [104]. In a trial comparing the absorption rate of catechin derived from black and green teas, the authors concluded that the tea variety did not determine the bioavailability of these flavonols. Plasma kaempferol and quercetin levels were similar in both cases, implying that fermentation did not affect the availability of polyphenols from the matrix [105].

### 5.4. Mechanical Processing

#### 5.4.1. Peeling

Peeling is one of the necessary preparation steps for both industrial and domestic food processing. Peels of many fruits and vegetables contain higher concentrations of bioactive compounds than the rest of the fruit. Some of them are discarded as agro-waste and can be utilized as such for extracting these valuable compounds. For example, citrus peels are abundant in phenolic compounds, such as flavanones, flavones, flavonols, and anthocyanins [106]. Peels of some common fruits (apple, banana, mandarin, and nectarine) are sources of non-extractable polyphenols [107].

The effects of peeling on polyphenol content have been scarcely addressed in literature. In some cases, this preparation step does not considerably affect the food item’s antioxidant capacity, as it is consumed without the peel, as in the case of bananas and citruses. However, it should be noted that, for example, the white layer in citruses is abundant in polyphenols; thus, a higher degree of peeling and removal of this layer would also lead to a lower polyphenol content in the consumed fruit. In addition, some fruits and vegetables can be consumed either with or without peels; thus, the difference in the polyphenol levels can be significant.

Studies have mainly focused on determining the phenolic composition and antioxidant activity of peels. However, few studies have addressed the effect of peeling on dietary polyphenols. For example, a study investigating how different heat-treatment regimes influenced onion flavonoids (quercetin and kaempferol) showed that the preprocessing step (peeling and trimming) caused the greatest loss in flavonoid content in onions (up to 39%) [108]. As onions contain multiple layers, the authors peeled only one layer in some cases and peeled several layers in other cases, resulting in a significant decrease in flavonoid content. This decrease in the flavonoid content could be because 90% of quercetin can be found in the first and second layers in onions [109].

Peeling of fruits and vegetables prior to processing was also observed to diminish phenolic bioactives. For example, peach puree containing peel tissues that underwent blanching and pasteurization had 7–11% higher antioxidant activity (measured by the β-carotene/linoleic acid assay) compared with the puree without the peel [110]. In another study, peeling caused a 13–48% loss in total phenolics in peaches, ranging between 316 and 397 mg kg^−1^ in peeled samples and between 376 and 609 mg kg^−1^ in unpeeled samples [111].

In the case of wine fermentation, there is also a big difference in whether the process is obtained with or without grape skin. Two white wine varieties were subjected to maceration at 5 °C for 24 h and further fermented with Saccharomyces bayanus BC commercial yeast. The authors reported that the total polyphenol index (representing the level of total polyphenols) and total flavonoids increased with an increase in alcohol content and the degree of fermentation. Higher increases were observed in the samples that contained skins, probably owing to a higher degree of polyphenol extraction [112].

#### 5.4.2. Grinding

Grinding is a processing technique to reduce the size of solid particles, using mechanical forces [113]. It has been applied in the preparation of fruit and vegetable powders, spices, tea, and coffee, as well as flour production. Most studies that assessed how grinding affected polyphenol content in the raw food material showed that the extraction of these antioxidants was mostly enhanced. However, this largely depends on the particle size of the powder end-product.

Grinding of *Hieracium pilosella* L. (an invasive weed famous for its antiseptic, antibacterial, and anti-inflammatory properties) yielded powders with enhanced antioxidant capacity, which was followed by an increase in the content of identified flavonoids and phenolic acids. However, this was observed only in the case of two fractions, which implies that the granulometric class needs to be optimal to obtain the highest degree of extraction of bioactive compounds and their corresponding activity [114]. Another study explored the difference in phenolic content between three types of coffee: Turkish, Espresso, and American coffee. In the case of American and Espresso coffee, fine–coarse powder exhibited the highest total polyphenol content and corresponding antioxidant activity, while in the case of Turkish coffee, fine–coarse and coarse powders were similar. When comparing these three coffees, which differ according to the brewing method, Espresso exhibited the highest antioxidant content and capacity, followed by Turkish and American coffee [115]. In tea, the reduction in the particle size of green tea leaves led to a higher decrease in almost all catechins measured [116].

However, in some cases, the granulometric characteristics do not influence the degree of bioactive extraction caused by grinding. For example, superfine grinding of green propolis enhanced the extraction of bioactive compounds, resulting in increased total phenolic content and antioxidant activity, regardless of the particle size [117].

An overview of studies investigating the effects of the main food-processing techniques (type, procedure, food matrix, and investigated polyphenol), and their effects on polyphenols content and bioavailability/bioaccessibility were presented in Appendix A (Table A1 and Table A2) respectively.

### 5.5. Emerging Food-Processing Technologies

Other emerging food-processing technologies, such as pulsed electric field, ultrasound treatments, high-pressure, and pulsed-light processing, have been developed in order to enhance the nutritive value, sensory properties, safety and preservation of several food components, including the improvement of polyphenol bioavailability in fruits and vegetables [45,118]. Through several physical and biochemicals changes of food components, these technologies contribute in reducing some food-related factors that impede polyphenols digestion, improving therefore their bioaccessibility and bioavailability [119]. The magnitude of these changes of the physicochemical and functional properties of food bioactive ingredients depends on the processing technology, the process parameters/conditions, and the food matrix [119,120]. Since we focused primarily on conventional food processing used at both domestic and industrial settings, these emerging technologies are beyond the scope of this review. However, it is important to investigate further their potential beneficial effects on dietary phytophenols in future studies and reviews to optimize their processing conditions according to each food matrix in order to maximize their bioaccessibility, bioavailability and health benefits.

## 6. Conclusions

As a large body of research evidence strongly supports the use of polyphenol-rich foods in the primary prevention and management of various chronic diseases, estimating their bioavailability is of great importance to draw straightforward conclusions regarding their actual efficacy. Since many fruits and vegetables are required to undergo food preparation prior to consumption, the summarized data regarding its influence on polyphenol content and bioavailability provide valuable insights into the actual benefits that could be obtained after consumption. Among the reviewed treatments that apply thermal processing, boiling is frequently reported to cause the most severe degradation in polyphenol content and bioavailability, mainly owing to their extensive leaching from the food matrix into the surrounding polar medium. Other treatments that utilize heat (such as canning and drying) have reported variable results. In canning, the presence of the surrounding medium plays an important role, while the intensity of the temperature regime and the duration of the process influence the end polyphenol content both when canning and drying are applied. Fermentation is reported to mainly lead to desirable changes in polyphenol content in food; however, bioavailability studies do not support the importance of fermentation in wine and black tea. Pickling can also yield products with an improved polyphenol profile, and studies have highlighted that this effect was mostly observed after an adequate fermentation period. Freezing and chilling are preservation techniques that are generally considered to produce minimal alterations in dietary polyphenols. Grinding is applied to facilitate the extraction of bioactive compounds, and this was mainly observed in polyphenols, with the particle size influencing their content. Data on the polyphenol-related effects of peeling are scarce; however, the existing evidence suggests that removing external layers can reduce the levels of polyphenols (and supposedly their bioavailability) in fruits and vegetables that contain polyphenol-rich peels (such as in the case of citruses). Although the bioavailability of polyphenols has gained popularity in the research community during the last decade, few studies have addressed this issue with regard to their bioavailability as influenced by food processing.

## Data Availability

Data sharing not applicable.

## Appendix A

**Table A1:** Overview of studies investigating the food-processing effects on polyphenols content and antioxidant activity.

Food Processing	Procedure	Food Matrix	Investigated Polyphenols	Effects on Polyphenols Content and/or Antioxidant Activity	Reference
Thermal Processing					
**Heat treatment**	Boiling for 60 min	Onions and asparagus	Flavonols	Flavonols decreased by 20.6% in onions and by 43.9% in asparagus Antioxidant activity decreased by 10.65%.	[64]
	Cooking in smaller and larger amounts of water	Zucchini, beans, carrots, potatoes	Rutin (zucchini), rutin and quercitrin (beans), chlorogenic acid (carrots), caffeic acid (potatoes)	Cooking in small water volumes caused a decrease by 11.1% in rutin (zucchini), by 1.8% in rutin (beans), by 0.9% in quercetin (beans), by 25.4% in chlorogenic acid (carrots), and by 38.6% in caffeic acid (potatoes). Cooking in larger water volumes caused a decrease by 30.6% in rutin (zucchini), by 29.7% in rutin (beans), by 26.7% in quercetin (beans), by 62.8% in chlorogenic acid (carrots) and by 42.3% in caffeic acid (potatoes).	[66]
	Cooking 15 min at 100 °C	Cherry tomato	Naringenin and chlorogenic acid	NS difference in meals containing raw and cooked tomatoes, however, the content of analyzed polyphenols was higher in those with raw tomatoes.	[72]
	Microwave (900 W) cooking for 12 or 20 min	Purple carrots	Acylated and non-acylated anthocyanin content	Decrease in both acylated (by 22.93%) and non-acylated (by 22.52%) anthocyanin content.	[75]
	Pasteurization (HTST—90 °C for 30 s and PEF 4 µs bipolar pulse with an electric field strength of 35 kV/cm and a frequency of 1200 pulses per second)	Apple juice	TPC	TPC after HTST pasteurization decreased by 32.3%. TPC after PEF pasteurization decreased by 14.49%.	[71]
	Oven-roasting (180 °C/15 min and 200 °C/30 min); frying (180 °C/4 min and 180 °C/8 min); microwaving (450 W/4 min and 750 W/4 min); boiling (for 30 and 60 min)	Onions	Flavonols and anthocyanins	Quercetin diglucoside decreased after all treatments. Quercetin monoglucoside decreased after microwaving and boiling and increased after frying and oven-roasting. All anthocyanins (cyanidin 3-glucoside, cyanidin 3-laminaribioside, cyanidin 3-(6″-malonylglucoside), cyanidin 3-(6″-malonyl-laminaribioside) decreased after all treatments.	[65]
	Blanching 2.5 min at 96–98 °C and cooking 10 min at 100 °C	Kale leaves	Quercetin, kaempferol, caffeic acid, *p*-cumaric acid, sinapic acid, ferulic acid, total polyphenols	All analyzed compounds decreased, with more severe decrease observed in the case of cooking. Antioxidant activity decreased by 32.6% in steamed, and by 45.45% in cooked kale leaves.	[68]
	Boiling, steaming, and microwaving (700 W) for 5, 10, and 15 min	Eggplants (4 varieties)	TPC	TPC Increased in all samples. Antioxidant activity increased in all samples.	[69]
	Baking (the baked product used 34 g of frozen blueberries as a polyphenol source; the comparison was carried out with a drink made by dissolving the same amount of frozen blueberries in water)	Blueberries	Total polyphenols, total anthocyanins, total procyanidins, quercetin, chlorogenic acid, caffeic acid, ferulic acid	Total polyphenols decreased but n.s. Total anthocyanins decreased significantly by 42.2% Total procyanidins increased but n.s. Quercetin increased but n.s. Chlorogenic acid increased significantly by 23.46% Caffeic acid increased but n.s. Ferulic acid increased but n.s.	[76]
	Tomato-sauce production (cooking for 60 min at 99 °C and crushing)	Tomato	Phenolic acids, flavanones and flavonols	Phenolic acids decreased by 43.18% Flavanones increased by 245.03% Flavonols increased by 17.21%	[73]
	Boiling, steaming, and microwaving	Cassava	Total extractable polyphenols	Total extractable polyphenols: increased by 152%, 236% and 164% after boiling, steaming and microwaving, respectively. Antioxidant activity: increased by 151.56%, 208.60% and 173.44% after boiling, steaming and microwaving, respectively.	[74]
	Sterilization until the temperature reached 123 °C	Runner bean	TPC	TPC decreased by 29.63% and 45.68% after 4 months and 12 months of storage, respectively. Antioxidant activity decreased by 87.89% and 89.47% after 4 months and 12 months of storage, respectively.	[70]
	Cooking in water (10 min), steaming (15 min, 97 ± 2 °C), microwaving (5 min, 800 W), and baking (15 min, 200 °C)	Sweet potatoes (2 varieties grown in Slovakia and Croatia)	TPC, TAC and phenolic acids (chlorogenic, neochlorogenic, and *trans*-ferulic)	TPC increased after almost all treatments and all varieties except in Beauregard from Croatia. TAC increased in all samples. chlorogenic acid decreased except after steaming in Beauregard from Slovakia. Neochlorogenic acid decreased in all samples. *Trans*-ferulic acid decreased in almost all samples except in boiled 414-purple from Croatia. Antioxidant activities increased in all samples.	[67]
**Canning**	Canning in syrup with thermal treatment	Cherries	TAC	TAC decreased by 44.89% Antioxidant capacity decreased by 33.03% measured by FRAP assay.	[78]
	Canning in 5% brine	Peanuts	*Trans*-resveratrol and three conjugates, caffeic acid, luteolin, genistein, quercetin and catechin	All measured compounds (except genistein) decreased in all samples.	[80]
	Canning—industrial vs. domestic thermal processing	Apricots	TPC, procyanidins, phenolic acids	TPC decreased by 13–47% in industrial setting and decrease by 2–33% in domestic setting Procyanidins decreased by 44% in industrial setting, and decreased by 2.4% in domestic setting Phenolic acids: higher decrease was observed in industrial vs. domestic cooking	[79]
	Canning of apricot pulps, followed by exhaustion under steam, and finally processed in an autoclave at 121 °C for 30 min	Apricots	Chlorogenic, neochlorogenic acid, catechin, kaempferol, quercetin, procyanidin B2	Chlorogenic acid increased by 31.31%, Neochlorogenic acid increased by 19.06%, Catechin increased by 37.19%, Kaempferol increased by 35.58%, Quercetin increased by 26.08%, Procyanidin B2 increased by 26.16% **The presented changes refer to only one variety, while two more varieties were used in the study.*	[121]
	Canning vs. freezing (−18 °C) vs. drying (65 °C)	Apricots	Ellagic acid, gallic acid, ferulic acid, epicatechin, epigallocatechin, rutin	Ellagic acid, gallic acid, ferulic acid, epicatechin and epigallocatechin were all affected by the treatments in the following order: canning yielded the highest content, followed by freezing and drying (in all three varieties). Canning yielded the highest content in rutin, followed by freezing and drying in one variety, while in other two varieties the rutin content was the highest in the case of canning, followed by drying then freezing.	[122]
**Drying**	Drying at 55 and 75 °C	Apricots (two cultivars)	Neochlorogenic, chlorogenic acid, catechin, epicatechin, rutin, quercetin-3-*O*-glucoside	Neochlorogenic and chlorogenic acid and catechin—decreased (higher decrease after 55 °C). Epicatechin, rutin and quercetin-3-0-glcoside—decreased (higher decrease after 75 °C). Antioxidant activity increased in one variety with higher increase after 75 °C drying.	[84]
	Drying (31–34 °C) for 8 days	Figs (2 varieties)	Total proanthocyanidin content	Total proanthocyanidin content increased in yellow figs And decreased in purple figs. Antioxidant activity (ABTS, DPPH, and FRAP assays) decreased in both varieties.	[88]
	Convection-oven-drying, freeze-drying (FD), microwave-drying, and air-drying with the sun exposure and without the sun exposure	Spearmint	TPC, hydroxycinammic acid derivatives	TPC—FD gave the highest TPC, followed by air with sun exposure, without sun exposure, microwave-drying and finally convection-drying. Caffeic acid—FD gave the highest content, followed by air-drying with and then without sun, convection-oven-drying and microwave-drying. Antioxidant activity: FRAP—the highest value in FD sample, followed by air-drying with sun, without sun, convection-drying, and microwave-drying; DPPH—the highest value in FD sample, followed by air-drying with sun, without sun, while microwave-and oven-drying gave similar values.	[123]
	Convective hot-air-drying at 65 and 80 °C vs. freeze-drying (FD)	Murtilla fruit	TPC, TAC, gallic acid, catechin, quercetin-3-glucoside, myricetin, kaempferol, quercetin	TPC—decreased by 37.33% after 65 °C, increased by 27.24% and 90.77% after 80 °C and FD, respectively. TAC—decreased by 38.46% and 66.67%, respectively, after 65 °C and 80 °C, and increased by 71.79% after FD. Gallic acid increased in all samples Catechin increased in 80 °C and FD, decreased in 65 °C Quercetin 3-β-d-glucoside, myricetin, kaempferol—decreased in all samples Quercetin—increased in 65 °C and 80 °C, decreased in FD Antioxidant activity—increase in all samples	[124]
	Drying (70 °C, 36 h)	Tomatoes	TPC, TFC	Rutin apioside, rutin, naringenin chalcone, chlorogenic acid—decrease Naringenin—was not identified in raw, but it was in dried tomato Antioxidant activity—increase in antioxidant capacity (ABTS, DPPH, CUPRAC assays)	[87]
	Hot-air-oven-drying or freeze-drying (FD)	Pumpkin flower	Free, bound, total phenols	Free phenols—similar values in case of oven-drying and FD, bound and total phenols—higher values after oven-drying. Antioxidant activity—hot-air-oven-dried pumpkin flours had slightly higher antioxidant activities than FD pumpkin flour samples.	[89]
	Drying at 60, 70, and 80 °C, respectively, at relative humidity level of 50% for heating time ranging from 0 to 40 h	Cocoa beans	TPC	Maximum TPC at 70 °C, and it decreased with the increased drying time.	[85]
	Drying at 40, 50, and 60 °C	Cocoa beans	TPC	TPC decreased by 45% after drying at 40 °C. Higher decrease with higher temperature.	[83]
	Freeze-drying (FD), drying at 50 °C for 48 h, 65 °C for 20 h, or 130 °C for 2 h until a moisture 89 content below 15% was obtained	Berries	Anthocyanins (individual), TPC	Anthocyanins—FD preserved them the best, 130 °C drying degraded them. TPC—highest values obtained after 65 °C drying, followed by FD, 50 °C and 130 °C drying. Antioxidant activity: ABTS•+ radical scavenging activity and ferric-reducing power varied depending on the berry type—raspberry and boysenberry showed the highest ABTS in the case of 65°C drying and redcurrant and blackcurrant in the case of FD, the same was observed for ferric-reducing power	[86]
	Vacuum/microwave-drying (480, 120 W), hot-air-drying (70, 60, 50 °C), and combined methods, such as pre-drying and finish-drying (60 °C + 480/120 W)	Jujube fruits (3 varieties)	TPC	TPC decreased in all samples; the lowest decrease was observed in the lowest temperature regime and in the combined process. Antioxidant activity decreased in ORAC values after drying, higher decrease in the case of higher temperature; lower decrease in the case of microwave-drying. Combined treatment gave lower decrease than hot-air-drying.	[82]
	Drying at 65 °C	Apricots	Chlorogenic, neochlorogenic acid, catechin, kaempferol, quercetin, procyanidin B2	Chlorogenic acid decreased by 16.02% Neochlorogenic acid decreased by 2.52% Catechin decreased by 3.66% Kaempferol decreased by 9.20% Quercetin decreased by 11.80% Procyanidin B2 decreased by 14.57% **The presented changes refer to only one variety, while two more varieties were used in the study.*	[121]
**Cold Processing**					
**Freezing/** **Chilling**	Freezing at −30 °C	Red Raspberries	Total phenolics, anthocyanins, lambertianin C, sanguiin H-6, ellagic acid	There was n.s. change in all analyzed compounds.	[91]
	Individual quick-freezing process	Berries	TPC, total monomeric anthocyanins	There was n.s. change in all analyzed compounds.	[94]
	Freezing	Blanched/cooked kale leaves	TPC	TPC decreased by 3%. Antioxidant activity decreased by 7%	[68]
	Cooling at 5 °C in the refrigerator, or freezing at −20 °C	Maqui fruits	Polyphenol and anthocyanin concentration	Polyphenol and anthocyanin increased in both cases, with higher increase in the case of frozen samples.	[92]
	Freezing by immersion at liquid nitrogen and freeze-drying at −50 °C	Apples	TPC	TPC decreased before digestion in both freezing methods. Antioxidant activity decreased before and after digestion (ABTS, CUPRAC, and FRAP assays).	[95]
	Slow vs. quick freezing	Strawberries	TPC and total monomeric anthocyanin content	TPC and total monomeric anthocyanin content increased in the case of quick-frozen samples compared with slow frozen samples.	[93]
	Deep-freezing to −18 °C	Apricots	Chlorogenic, neochlorogenic acid, catechin, kaempferol, quercetin, procyanidin B2	Chlorogenic acid increased by 6.55% Neochlorogenic acid increased by 1.08% Catechin increased by 17.69% Kaempferol increased by 15.95% Quercetin increased by 4.35% Procyanidin B2 increased by 7.95% **The presented changes refer to only one variety, while two more varieties were used in the study.*	[121]
	Freeze-drying vs. hot-air-drying vs. infrared-drying vs. pasteurization of apple puree)	Red-fleshed apples	Phenolic acids, flavan-3-ols, flavonols, anthocyanins, flavanones, dihydrochalcones	Compared with the freeze-dried snack: Infrared-drying caused important losses in most of the apple (poly)phenolics Purée pasteurization maintained 65% the (poly)phenols Hot-air-drying maintained 83% the (poly)phenols Anthocyanins were degraded to a higher extend after all thermal processing technologies.	[125]
**Biochemical Processing**					
**Fermentation**	Fermentation of red grape	Red wine, dealcoholized red wine, and red grape juice	Malvidin-3-glucoside (M-3-G)	M-3-G—red wine (68 mg), dealcoholized red wine (58 mg) and red grape juice 117 mg	[103]
	Black-tea fermentation	Black vs. green tea	Quercetin, kaempferol	Eight cups of black tea (4 g tea solids) provided 108 µmol of quercetin glycosides (equivalent to 32.5 mg as free quercetin) and 72 µmol of kaempferol glycosides. The green tea (4 g tea solids) provided 104 µmol of quercetin glycosides and 58 µmol of kaempferol glycosides per day.	[105]
	Fermentation (red-wine production)—comparison between red wine and red grape juice	Red grape	Anthocyanins	Total anthocyanin content was almost equal in both red grape juice and red wine.	[102]
	Fermentation to apple cider	Apple (5 varieties)	TPC, catechin, caffeic acid	TPC decreased only in two varieties. Catechin increased in all varieties Caffeic acid increased in two varieties	[101]
	Natural fermentation	Legumes—pigeon pea, bambara groundnut, African yam bean, and kidney bean	Free and bound soluble phenol content	Free soluble phenol content increased, Bound phenol content decreased Antioxidant activity: free soluble phenolic compounds of fermented legumes had increased reducing power, free radical scavenging ability, and inhibition of lipid peroxidation compared with unfermented legumes.	[100]
	Fermentation with naturally present bacteria and with lactic acid bacteria	Eight legumes: black cow gram, mottled cowpea, speckled kidney bean, lentil, small rice bean, small runner bean and two soya beans	TPC	TPC increased in almost all samples Antioxidant activity increased in mottled cowpea, speckled kidney bean and small rice bean, and n.s. changes in all other samples.	[99]
	Fermentation with *Aspergillus awamori*	Millet	TPC	TPC—increase (more than 2-fold) Antioxidant activity (measured both by DPPH and ABTS assays)—slight increase by 3.75% and by 2.12%	[98]
	Ting fermentation (at different time and temperature regimes)	Sorghum	TPC, total flavonoid and total tannin content	All analyzed compounds—optimal values at 27 °C for 72 h Antioxidant activity—the best one was obtained in a sample fermented at 27 °C for 24 h	[97]
	Lactic acid fermentation (milk enriched with dates)	Two types of yoghurt enriched with dates	TPC and antioxidant activity	TPC: 34 and 37 mg of GAE 100 g^−1^ for, respectively, yogurt made with dates blended with milk and yogurt produced using small pieces of dates that were added to milk. Antioxidant activity (measured by DDPH) was 51% and 57% for yogurt made with dates blended with milk and yogurt produced using small pieces of dates that were added to milk, respectively.	[126]
**Pickling**	Pickling (with variable salinity and the addition of *Lactobacillus plantarum*)	Potherb mustard	The total free phenolic acids, the total phenolic acids, total phenolics	Total free phenolic acids increased. Total phenolics and total phenolic acids decreased. Antioxidant activity decreased by 35%.	[127]
	Pickling	Papaya	TPC, TFC	TPC decreased by 68.40% TFC decreased by 65.90% Antioxidant activity (DDPH assay) decreased.	[128]
	Pickling	Green beans, green pepper, chili pepper, white cabbage, cauliflower, cucumber, sneak melon, tomato, carrot, garlic	TPC	TPC—after 15 days—decreased in all vegetable; after 30 and 60 days increased in all vegetables. Antioxidant activity: the Trolox equivalent antioxidant capacity decreased after 15 days, then increased after 30 and 60 days for green beans, green pepper, chili pepper, cauliflower, white cabbage, cucumber and sneak melon. For tomato, carrot and garlic it increased during all time points. DPPH assay: RSA decreased followed by increase in all except green pepper, cauliflower, cucumber and sneak melon which decreased during all time points.	[129]
	Pickling	Soybeans	TPC, TPA content, TFC, naringenin, and vanillin	TPC and TFC increased by 47% and 42%, respectively. TPA decreased by 35% Naringenin and vanillin increased (24 and 2.5-fold, respectively).	[130]
**Mechanical Processing**					
**Peeling**	Peeling, trimming and chopping	Onions	Flavonoids (quercetin and kaempferol)	Flavonoids decreased by 39%.	[108]
	Removal of periderm material in purée processing	Peaches	TPC, chlorogenic, neochlorogenic acid, catechin, caffeic acid	TPC—minor differences between the samples with and without the peel. Chlorogenic and neochlorogenic acid decreased in puree with peel (vs. puree without peels) Catechin increased in samples with peels (vs. puree without peels). Caffeic acid—slight decreased slightly in puree with peels (vs. puree without peels). Antioxidant activity: Peach puree with periderm tissue that underwent blanching and pasteurization had 7–11% higher antioxidant activity (measured by beta-carotene/linoleic acid assay).	[110]
	Peeling	Clingstone peaches	TPC	TPC decreased by 13–48%	[111]
	Fermentation with *Saccharomyces bayanus BC* (with and without skins)	White grape	Total polyphenol index, total flavonoids	Total polyphenol index increased especially in grapes with skins. Total flavonoids decreased in samples without skins, while they increased in samples with skins.	[112]
**Grinding**	Superfine grinding (included sieving through a 180 µm sieve once, or for 20–120 min)	Green tea	Catechins	Catechins decreased in all analyzed catechins Antioxidant activity increased in OH scavenging rate between the sample that was once sieved through a 180 µm sieve and those that underwent sieving for 20–120 min.	[116]
	Grinding and sieving	*Hieracium pilosella* L.	Flavonoids and phenolic acids	Increase in luteolin-7-glucoside, umbelliferone, luteolin, 3,5-dicaffeoylquinic acid, and chlorogenic acid (the maximum values in the case of 180–315 and 315–500 µm). Antioxidant activity increased in sieved samples (the maximum activity obtained at 180–315 and 315–500 µm).	[114]
	Grinding to produce American, Turkish, and Espresso coffees	Coffee beans	TPC	TPC—the highest observed in the case of fine–coarse powder. Further grinding decreased TPC. Antioxidant activity—the highest observed in the case of fine–coarse powder. Further grinding decreased the antioxidant activity (DPPH assay).	[115]
	Superfine grinding method	Brazilian green propolis	TPC	TPC increased (regardless of the particle size) Antioxidant activity increased (measured by ABTS and DPPH, regardless of the particle size)	[117]

**Abbreviations:** TPC—total phenolic content, TAC—total anthocyanin content, TPA—total phenolic acid content; TFC—total flavonoid content, HSTS—high-temperature short-time; PEF—pulsed electric field; sig—significant, n.s.—non significant; DPPH—2,2-diphenyl-1-picrylhydrazyl; ABTS—2,2’-azino-bis(3-ethylbenzothiazoline-6-sulfonic acid; FRAP—ferric reducing antioxidant power; CUPRAC—cupric-reducing antioxidant capacity; GAE—gallic acid equivalent; RSA—radical scavenging activity.

**Table A2:** Overview of studies investigating the food-processing effects on polyphenols bioavailability/bioaccessibility.

Food Processing	Procedure	Food Matrix	Investigated Polyphenols	Type of Study	Effects on Polyphenols on Bioavailability/Bioaccessibility	Reference
**Heat treatment**	Cooking 15 min at 100 °C	Cherry tomato	Naringenin and chlorogenic acid	In vivo crossover human study on 5 subjects	Plasma naringenin increased 2 h after consumption Plasma chlorogenic acid increased 2 and 6 h after consumption	[72]
	Microwave (900 W) cooking for 12 or 20 min	Purple carrots	Acylated and non-acylated anthocyanin content	In vivo crossover human study, 12 subjects	Acylation of anthocyanins: 11–14-fold decreased in anthocyanin recovery in urine and an 8–10-fold decreased in anthocyanin recovery in plasma. Cooking increased the recovery of non-acylated anthocyanins but not acylated anthocyanins.	[75]
	Baking by using 34 g of frozen blueberries as a polyphenol source and comparison with blueberry drink.	Blueberries	Total polyphenols, total anthocyanins, total procyanidins, quercetin, chlorogenic acid, caffeic acid, ferulic acid	In vivo crossover human RCT, 10 subjects	AUC of phenolic metabolites were compared between baked product and blueberry drink—increase in m-hydroxyphenylacetic, ferulic, isoferulic, and hydroxyhippuric acids and decrease in hippuric, benzoic, salicylic, and sinapic acids.	[76]
	Tomato sauce production (boiling for 60 min at 99 °C and crushing)	Tomato	Phenolic acids, flavanones and flavonols	In vivo crossover human RCT with 8 subjects	Increase of plasma concentration and urinary excretion of naringenin glucuronide.	[73]
	Thermal treatment at 90 °C for 60 s	Orange, kiwi, pineapple and mango juices	Total phenolic acids, total flavonoids, TPC	In vitro gastrointestinal digestion model	Bioaccessability of total phenolic acids decreased by 12.70% compared with the control sample Bioaccessability of total flavonoids increased by 2.65% compared with the control sample Bioaccessability of TPC decreased by 4.17% compared with the control sample	[131]
	Heating at 80 and 90 °C for 30 s	Apple, orange and grape juice	Total Polyphenols	In vitro gastrointestinal digestion model	Bioaccessibility of total polyphenols in heated apple juices did not change compared to the control juice. Bioaccessibility of total polyphenols in grape juice increased by 33.9% and 27.3% for those treated at 80 and 90 °C, respectively. Bioaccessibility of total polyphenols in orange juice increased by 19% and 29.2% for samples heated at 80 and 80 °C, respectively.	[132]
	Boiling, steaming, microwaving	Cassava	Total extractable polyphenols	In vitro gastrointestinal digestion model	Bioaccessability for total extractable polyphenols was 72.94% after boiling, 74.54% after steaming, and 72.67% after microwaving Bioaccessability for antioxidative activity was 34.78% after boiling, 36.71% after steaming and 36.85% after microwaving	[74]
**Drying**	Drying (31–34 °C) for 8 days	Figs (2 varieties)	Total proanthocyanidin content	In vitro simulated gastrointestinal digestion model	Increase in bioaccessibility of total proanthocyanidins and chlorogenic acid content and decrease in anthocyanin bioaccessibility.	[88]
	Drying (70 °C, 36 h)	Tomatoes	TPC, TFC	In vitro gastrointestinal digestion model	Compared with raw tomatoes, dried tomatoes had higher TPC and TFC values during digestion.	[87]
	Hot-air-oven-drying or Freeze-drying	Pumpkin flower	Free, bound, total phenols	In vitro digestion enzymatic extraction method	Phenolic bioaccessibility for oven-dried sample was 30.76% and for freeze-drying it was 29.19%.	[89]
	Freeze-drying vs. hot-air-drying vs. infrared-drying (35, 40, 50, and 60 °C) vs. pasteurization of apple puree	Apples puree	Total polyphenols	In vivo human crossover study, 3 subjects	Percentage of urine excretion of total polyphenols was the highest in the case of pasteurized puree, followed by hot-air-dried and then freeze-dried samples.	[125]
**Freezing**	Freezing by immersion at liquid nitrogen and freeze-drying at −50 °C	Apples	TPC	In vitro gastric digestion model	Decrease in TPC during and after digestion with both freezing methods.	[95]
	Individual quick freezing (IQF) and conventional freezing (CF)	Strawberries	TPC, TFC, TAC, TMAC	In vitro gastric digestion model	Overall, after the completion of in vitro digestion, bioaccessibility values for TPC, TFC, TMAC, and TAC were found to be 94–105%, 64–91%, 47–83%, and 55–84%, respectively. TMAC from frozen strawberries was significantly more bioaccessible than that of fresh strawberries (*P* < 0.05).	[133]
**Fermentation**	Fermentation of red grape	Red wine, dealcoholized red wine and red grape juice	Malvidin-3-glucoside (M-3-G)	In vivo, crossover human RCT, 6 subjects	The plasma levels of M-3-G was similar between all three arms.	[103]
	Black tea fermentation	Black vs. green tea	Quercetin, kaempferol	In vivo human study, 18 subjects	Quercetin and kaempferol plasma levels showed no difference between the two tea types.	[105]
	Fermentation of red wine and red grape juice	Wine vs. red grape juice	Anthocyanins	Acute in vivo human study, 9 subjects	Total dietary anthocyanins—higher bioavailability of those from red grape juice compared to those in red wine. Low urinary excretion of dietary anthocyanins with values below 1%. Only 0.25% and 0.18% of the administered dose of total anthocyanins was excreted within 7 h after red grape juice and red wine ingestion, respectively.	[102]
	Controlled alcoholic fermentation	Orange juice	A total of 24 (poly)phenol metabolites including both flavanone and phenolic acid derivatives	In vivo human crossover study, 9 subjects	Bioavailability of phenolic metabolites in urine in comparison with total intake of polyphenols was lower in fermented orange juice (around 46%) than in orange juice (59%).	[134]

**Abbreviations:** TPC—total phenolic content, TAC—total anthocyanin content, TPA—total phenolic acid content; TFC—total flavonoid content, RCT—randomized controlled trial, AUC—area under the ROC curve, M-3-G—malvidin-3-glucoside, FD—freeze-drying, TMAC—total monomeric anthocyanin content.
